# 

**Published:** 1903-07-15

**Authors:** 


### NATIONAL DENTAL ASSOCIATION.

Following is a partial list of clinics promised for the coming meeting in Asheville, N. C.

An excellent list of papers is being prepared by the various sections. From the preparations that are being made, the coming meeting will be an unusually interesting and profitable one.

### CLINICS.

1. Dr. Levi C. Taylor, Hartford, Conn.—"Hygienic Fillings." 2. Dr. S. Eldred Gilbert, Philadelphia, Pa.—"Sharp Seamless Crown Outfit." 3. Dr. R. C. Brophy, Chicago, Ills.—"Something in Porcelain Work." 4. Dr. Garrett Newkirk, Los Angeles, Cal.—"Advantages of the Hollow Post Combined with the Inlay Principle for Cantilever and Bridge Abutments." 5. Dr. D. O. M. Le Croon, St. Louis, Mo.—"Modern Porcelain Art, and Oil Colors, as Applied to Dental Prosthesis." 6. Dr. Wm. H. G. Logan, Chicago, Ills.—"Pyorrhea Alveolaris." 7. Dr. Howard T. Stewart, Memphis, Tenn.—"Partial Removal and Decalcification of Cementum in Treatment of Riggs' Disease." 8. Dr. F. Lee Hollister, Wilkesbarre, Pa.—"A Demonstration of the Application of Dr. Edward H. Angle's Fracture Bands in Fractures of Maxillæ, Superior and Inferior." 9. Dr. Geo. Evans, New York.—"The Cementation of Crowns and Bridges with Gutta-percha Cement." 10. Dr. J. H. Feagan, Spartansburg, S. C.—"Demonstrating the Advantages of an Improved Flask in Investing and Packing

Vulcanite Dentures." 11. Dr. Robert J. Cruise, Chicago, Ills.—"Administration of Nitrous Oxide with a New Nasal Inhaler." 12. Edwin C. Blaisdell, D.M.D., Portsmouth, N. H.—"The Use of Non-Cohesive Gold." 13. Dr. Russell Markwell, Galveston, Texas.—"Porcelain Crowns." 14. Dr. Paul W. Evans, Washington, D. C.—"Specimens of Porcelain Work and a Method of Making Seamless Gold Shell Crowns." 15. Dr. F. J. Capon, Toronto, Canada—"Porcelain Crowns, Sections and Inlays." 16. Dr. Alfred Owre, Minneapolis, Minn.—"Cavity Preparation in Natural Teeth." 17. Dr. Burton Lee Thorpe, St. Louis, Mo.—"A Method of Protecting the Cervical Margin in Cement Fillings." 18. Dr. D. J. McMillen, Kansas City, Mo.—"Combination Cohesive and Non-Cohesive Gold." 19. Dr. H. Herbert Johnson, Macon, Ga.—"An Improved Modification of the Richmond Crown." 20. Dr. R. Ottolengui, New York—"Models and Appliances Representing Artificial Vela and Obturators for Cleft Palate Cases." 21. Dr. Wm. Leon Ellerbeck, Salt Lake City, Utah—"Porcelain Fillings and Furnace Construction." 22. Dr. Chas. P. Pruyn, Chicago, Ills.—"Root-Canal Filling, Using Sandarac Varnish and Gold Wire Points." 23. Dr. Hart J. Goslee, Chicago, Ills.—To be announced. 24. Dr. Rudolph Beck, Chicago, Ills.—To be announced. 25. Dr. Wm. Reeves, Chicago, Ills.—"Porcelain Inlays." 26. Dr. Joseph Head, Philadelphia, Pa.—To be announced. 27. Dr. H. B. Tileston, Louisville, Ky.—"Gold Inlay (using Copper Amalgam Matrix.") 28. Dr. Harry P. Carlton, San Francisco, Cal.—To be announced. 29. Dr. W. A. Capon, Philadelphia, Pa.—"Porcelain." 30. Dr. L. E. Custer, Dayton, Ohio—"Porcelain." 31. Dr. B. Holly Smith, Baltimore, Md.—"Some Novel Attachments for Removable Bridge and Metal Plate Work." 32. Dr. W. E. Grant, Louisville, Ky.—"Abutments for Esthetic Crown and Bridge Work." 33. Dr. A. R. Begun, Des Moines, Iowa—"Some New Things in Gold Work." 34. Dr. Emory A. Bryant, Washington, D. C.—"Replaceable Facings for Crown and Bridge Work, and Repairs." 35.

Dr. Henry C. Raymond, Detroit, Mich.—"Porcelain." 36. Dr. Geo. W. Schwartz, Chicago, Ills.—"Method for Constructing a Continuous Gum, Upper Set of Teeth. Table Clinic." 37. Dr. C. Edmund Kells, New Orleans, will exhibit models showing his method of extracting impacted third molars; he will also give a demonstration in X-Ray Work. 38. Dr. Robert E. Payne, New York,—"Implantation." 39. Dr. Wm. K. Slater, Knoxville, Tenn.—"Porcelain Work." 40. Dr. Thos. P. Hinman, Atlanta, Ga.—"Porcelain Inlays." 41. Dr. L. C. Alexander, Charlotte, N. C.—"Gold Inlays." 42. Dr. J. Y. Crawford, Nashville, Tenn.—"Oral Clinics on the Management of Children's Teeth, also on Management of Mouths of very Aged People."

### ANNOUNCEMENTS.

At the thirty-ninth annual meeting of the Missouri State Dental Association, held at Kansas City, May 19-21, 1903, the following officers were elected: President, J. H. Kennerly, St. Louis; First Vice-President, F. W. Franklin, Kansas City; Second Vice-President, F. H. Achelpohl, St. Charles; Recording Secretary, H. H. Sullivan, Kansas City; Corresponding Secretary, S. T. Bassett, St, Louis; Treasurer, J. F. Fry, Moberly.

Place and time of next meeting: St. Louis, third Tuesday in May, 1904.

THE national meetings, the Faculties Association, Board of Examiners, and the National Dental Association begin their meeting July 24th, at 11 a. m., at Asheville, N. C. The railways have made the usual rate of a fare and a third for the round trip. There is also a regular tourist rate which is good for six months and is about the same. So that for those who wish to stay longer it would be just as well to get this regular tourist ticket. The tourist rate from Detroit by way of C. H. & D. and Queen & Crescent Route is $27.75. The fare and a third rate is only fifty cents less. Corresponding rates can be had from all other points. The best trains are the 12.35 noon, from Detroit which connects at Cincinnati with the evening 8.05 Queen & Crescent, which arrives at Asheville the next afternoon at 1.10.

